# Noninvasive Detection of *Echinococcus multilocularis* Tapeworm in Urban Area, Estonia

**DOI:** 10.3201/eid2101.140136

**Published:** 2015-01

**Authors:** Leidi Laurimaa, John Davison, Liivi Plumer, Karmen Süld, Ragne Oja, Epp Moks, Marju Keis, Maris Hindrikson, Liina Kinkar, Teivi Laurimäe, Jaana Abner, Jaanus Remm, Peeter Anijalg, Urmas Saarma

**Affiliations:** University of Tartu, Tartu, Estonia

**Keywords:** Echinococcus multilocularis, fox tapeworm, echinococcosis, alveolar echinococcosis, noninvasive molecular diagnostics, red fox, feces, zoonoses, Estonia

**To the Editor:** Alveolar echinococcosis, which is caused by the fox tapeworm *Echinococcus multilocularis*, is an emerging disease in Europe that shows a high mortality rate (*1*). Humans can become infected after ingesting parasite eggs (e.g., through direct contact with dogs and red foxes [*Vulpes vulpes*] or with their contaminated feces). *E. multilocularis* tapeworm eggs are extremely resistant and can remain viable in the environment for years (*2*).

Numbers of red foxes have increased in many countries in Europe in recent decades, and the *E. multilocularis* tapeworm has also expanded its range. This tapeworm has recently been reported in 17 countries in Europe, including Lithuania, Latvia, and Estonia (*1*). Foxes and associated tapeworms are also increasingly found in urban areas, prompting considerable public health concern (*1*,*3*). Foxes began to colonize urban areas in Estonia in 2005, and they have since been reported in 33 of 47 towns nationwide (L. Plumer, et al. unpub. data). Because ≈30% of foxes are infected with the *E. multilocularis* tapeworm in natural habitats in Estonia (*4*), it is essential to monitor parasite spillover into urban areas, where it could become a serious public health risk. Consequently, there is an acute need for methods that can effectively detect the parasite and thereby help prevent human infection.

Although immunologic (*2*) and genetic methods (*5*–*7*) are available for identifying *Echinococcus* spp. parasites, a sensitive molecular diagnostic method that detects tapeworms and identifies their host species from degraded fecal samples would be useful. The purposes of this study were to develop a sensitive, noninvasive, genetic method to identify the host species by discriminating between feces of red foxes and dogs; detect *E. multilocularis* tapeworms in feces and distinguish them from the related parasite *E. granulosus*; and collect carnivore feces in an urban area in Estonia to identify this tapeworm.

Fecal samples suspected to be from red foxes were collected during January–March 2012 and January–March 2013 from streets and grassy areas of Tartu, Estonia. Tartu is a relatively small city (area 39 km^2^) with 98,000 human inhabitants. We surveyed 14 transects, each ≈4 km in length, that included all major districts in the city (Figure). Each transect was searched weekly during the study period (total ≈850 km surveyed).

**Figure:**
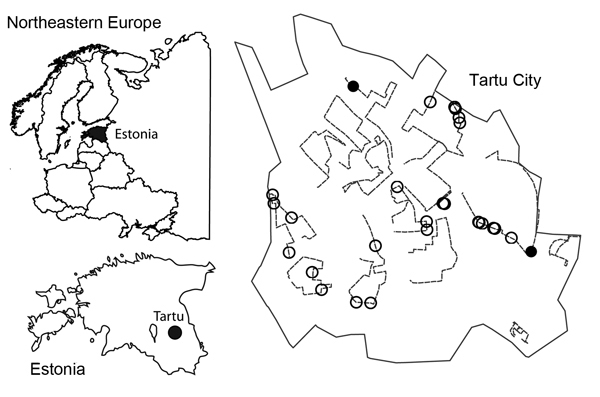
Location of Tartu in northeastern Europe, Estonia, and red fox feces sampling area in Tartu. The Tartu City boundary is indicated by a solid black line, survey transects are indicated by dashed lines, and fox fecal samples (n = 28) are indicated by circles. Filled circles (n = 2) indicate samples positive for *Echinococcus multilocularis* tapeworms.

A total of 137 fecal samples were collected and stored at −80°C for ≥1 week to avoid risk of infection from any *Echinococcus* spp. eggs present (*2*) because *E. multilocularis* (*4*) and *E. granulosus* (*8*,*9*) tapeworms have been found in Estonia. Samples of ≈250 mg were placed into 2-mL tubes, heated at 65°C for 15 min, and stored at −80°C. The heating and cooling procedure helps to break the parasite egg shells, enabling more efficient DNA extraction. DNA was extracted by using the QIAamp DNA Stool Mini Kit (QIAGEN, Hilden, Germany) according to the manufacturer’s instructions.

Species-specific primers (Technical Appendix Table) were designed to amplify short sequences of mitochondrial DNA. On the basis of primer specificity and amplicon size, we determined host and parasite species (Technical Appendix Figure 1). DNA extraction and PCR were performed in a laboratory dedicated to environmental samples (for a complete description of methods, see Technical Appendix).

DNA was successfully extracted and amplified from 119 (86.9%) of 137 fecal samples. Of usable samples, 28 (23.5%) were from red foxes and 91 (76.5%) were from dogs. Two fox fecal samples (7.1%; 95% binomial CIs 0.9%–23.5%) were infected with *E. multilocularis* tapeworms; none of the dog samples were infected.

To verify parasite identification, we amplified DNA from the 2 *E. multilocularis–*positive samples with *E. multilocularis*–specific primers and sequenced the amplification products. To verify host species identification, we used primers that produced longer amplification products (327 and 197 bp) than the corresponding PCR primers and sequenced amplification products from 5 fox samples and 5 dog samples.

Sequencing procedures were performed according to the methods of Saarma et al. (*10*). Sequences from both *E. multilocularis–*positive samples showed 100% identity with an *E. multilocularis* tapeworm sequence (GenBank accession no. AB018440) (Technical Appendix Figure 2). All sequenced fox and dog samples also belonged to the corresponding species.

To estimate the sensitivity of this noninvasive genetic method, we determined the number of *E. multilocularis* eggs necessary to obtain a positive PCR result (Technical Appendix Figure 3). One egg was sufficient to give an *E. multilocularis* tapeworm–specific result.

In summary, we developed a noninvasive genetic method that identifies *E. multilocularis* tapeworms and their host species in carnivore fecal samples found in urban environments. Furthermore, these tapeworms can even be detected in fecal samples from red foxes when only 1 parasite egg is present. Thus, this method is highly sensitive and discriminatory and can be used with degraded fecal samples to monitor *E. multilocularis* tapeworms and their hosts.

Technical Appendix Methods used to detect *Echinococcus multilocularis* tapeworms in red fox fecal samples in an urban area, Estonia.
