# Supplementary material for: Noninvasive Detection of Echinococcus multilocularis Tapeworm in Urban Area, Estonia
Source: Emerg Infect Dis. 2015 Jan;21(1):163–4. doi: 10.3201/eid2101.140136 (PMC4285250; doi:10.3201/eid2101.140136)
Supplement: Technical Appendix — Methods used to detect Echinococcus multilocularis tapeworms in red fox fecal samples in an urban area, Estonia. [file 14-0136-Techapp-s1.pdf]

Article DOI: http://dx.doi.org/10.3201/eid2101.140136

Noninvasive Detection of *Echinococcus multilocularis* Tapeworm in Urban Area, Estonia

Technical Appendix

Methods Used to Detect *Echinococcus multilocularis* Tapeworm in Red Fox Fecal Samples, Urban Area, Estonia

Methods

DNA Purification from Fecal Samples

Samples were collected and stored at −80°C for ≥1 week to avoid the risk of infection with any *Echinococcus* spp. eggs present. Approximately 250 mg was placed into 2-mL tubes, heated at 65°C for 15 min, and stored at −80°C. The heating and cooling procedure helps to break the parasite egg shells, enabling more efficient DNA extraction. DNA was extracted by using the QIAamp DNA Stool Mini Kit (QIAGEN, Hilden, Germany) according to the manufacturer's instructions.

PCR

Species-specific primers (online Technical Appendix Table) were designed to amplify short sequences of mitochondrial DNA. The *Echinococcus multilocularis* tapeworm–specific primer pair (EMfor1 and EMrev1) amplifies tRNA-Ile/Lys, the *E. granulosus* tapeworm–specific (EGfor1 and EGrev1) and fox-specific (F1 and RVu) primer pair amplifies 12S rRNA, and the dog-specific (Dog1f and HW1r) primer pair amplifies part of the mitochondrial DNA control region. Although the 3 primers pairs were highly specific, the dog primers can potentially amplify gray wolf (*Canis lupus*) DNA. In other similar studies, only the parasite (*1*) or the host species (*2*) was detected, and much longer sequences were amplified, making analysis of degraded samples less sensitive. Quantitative real-time PCRs for detection and quantification of

E. multilocularis DNA in fox feces are available (*3*,*4*) but for many laboratories the cost of these PCRs limits their use.

PCR was performed twice for each sample in a volume of 20 µL containing 10× Advantage 2-SA Buffer and 50× Advantage2-Polymerase Mix (Clontech, Mountain View, CA, USA), 0.2 µmol/L dNTP (Fermentas, Waltham, MA, USA), 0.25 µmol/L of each primer, and 10 µL of purified DNA. The PCR conditions were 95°C for 1 min; 10 cycles at 95°C for 20 s, annealing at 68°C for 20 s (temperature reduced by 0.5°C in each cycle) and extension at 68°C for 30 s; 35 cycles under the same conditions, except that the annealing temperature was 63°C; and a final extension at 68°C for 3 min. PCR products were separated by electrophoresis on 2.5% agarose gels. DNA extraction and PCR was performed in a laboratory dedicated to environmental samples.

Sensitivity Assay for Detecting *E. multilocularis* Tapeworm in Red Fox Feces

To estimate the sensitivity of the noninvasive genetic method, we determined the number of *E. multilocularis* eggs necessary to obtain a positive PCR result. We analyzed 1, 3, 5, 7, 10, 15, and 20 fox tapeworm eggs, which were added to fox fecal samples (≈250 mg) that were previously known to be uninfected with these eggs. The mixture was heated and deep frozen before DNA extraction, and PCR was performed as described above. The assay was repeated three times. We found that 1 egg was sufficient to give an *E. multilocularis* tapeworm–specific result (online Technical Appendix Figure 3). In other studies, flotation methods for concentrating parasite eggs and alkaline lysis of egg shells have been used before PCR analysis (*5*,*6*). This study demonstrates that concentrating eggs is unnecessary when applying the method described above.

References

1. Trachsel D, Deplazes P, Mathis A. Identification of taeniid eggs in the faeces from carnivores based on multiplex PCR using targets in mitochondrial DNA. Parasitology. 2007;134:911–20. PubMed http://dx.doi.org/10.1017/S0031182007002235

2. Nonaka N, Sano T, Inoue T, Armua MT, Fukui D, Katakura K, et al. Multiplex PCR system for identifying the carnivore origins of faeces for an epidemiological study on *Echinococcus multilocularis* in Hokkaido, Japan. Parasitol Res. 2009;106:75–83. PubMed http://dx.doi.org/10.1007/s00436-009-1629-0

3. Dinkel A, Kern S, Brinker A, Oehme R, Vaniscotte A, Giraudoux P, et al. A real-time multiplex-nested PCR system for coprological diagnosis of *Echinococcus multilocularis* and host species. Parasitol Res. 2011;109:493–8. PubMed http://dx.doi.org/10.1007/s00436-011-2272-0

4. Knapp J, Millon L, Mouzon L, Umhang G, Raoul F, Ali ZS, et al. Real time PCR to detect the environmental faecal contamination by *Echinococcus multilocularis* from red fox stools. Vet Parasitol. 2014;201:40–7. PubMed http://dx.doi.org/10.1016/j.vetpar.2013.12.023

5. Mathis A, Deplazes P, Eckert J. An improved test system for PCR-based specific detection of *Echinococcus multilocularis* eggs. J Helminthol. 1996;70:219–22. PubMed http://dx.doi.org/10.1017/S0022149X00015443

6. Al-Sabi' MN, Kapel CM, Deplazes P, Mathis A. Comparative copro-diagnosis of *Echinococcus multilocularis* in experimentally infected foxes. Parasitol Res. 2007;101:731–6. PubMed http://dx.doi.org/10.1007/s00436-007-0537-4

Technical Appendix Table. Mitochondrial DNA primers used for PCR detection of *Echinococcus multilocularis* fox tapeworm, Estonia

Primer*	Sequence, 5′→3′	Amplicon size, bp	Specificity†
EMfor1	TGATTAATAGGGCTGCATG	120	Fox tapeworm
EMrev1	CACATTACTGAGGTAAGAAC	120	Fox tapeworm
F1	CCATGAAGCACGCACACA	76	Red fox
RVu	GTTAATATGTTTATGGCCTCA	76	Red fox
EGfor1	GTGTGTTACATTAATAAGGGTG	149	Dog tapeworm
EGrev1	CTTGTTACGACTTACCTCAA	149	Dog tapeworm
Dog1f	GTATCTCCAGGTAAACCCTTCTC	56	Domestic dog
HW1r	CAAACCATTAATGCACGACGT	56	Domestic dog

*EM, *Echinococcus multilocularis*; for, forward; EG, *E. granulosis*; rev, reverse.
†Red fox, *Vulpes vulpes*; dog tapeworm, *E. granulosus*; domestic dog, *Canis familiaris*.

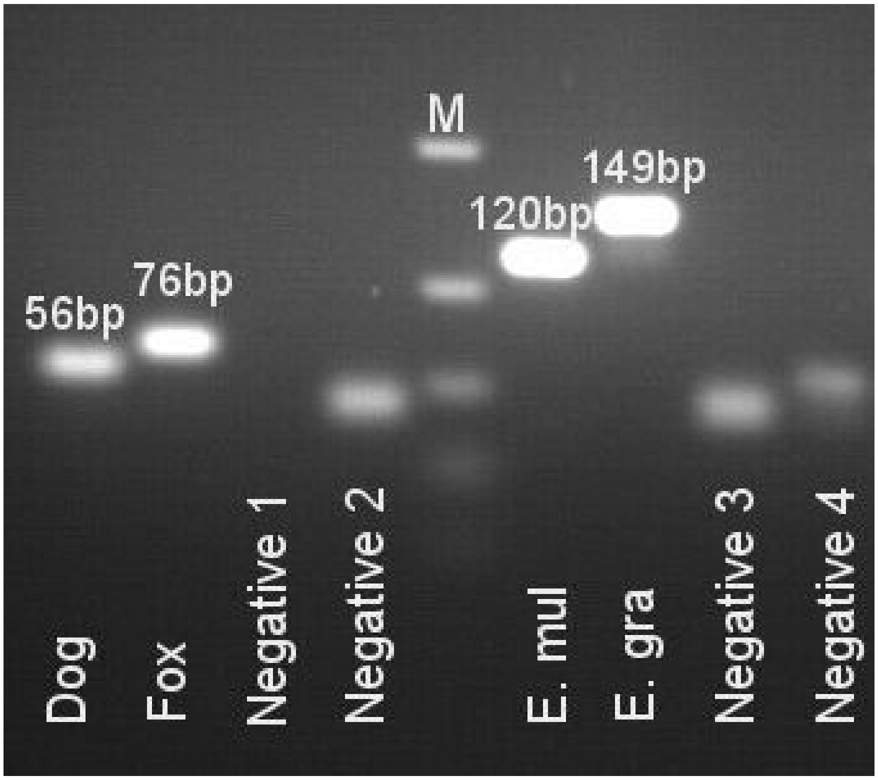

Technical Appendix Figure 1. Host and parasite species determination on the basis of primer specificity and amplicon size. PCR amplification of mitochondrial DNA from dog and red fox tissue and from *Echinococcus multilocularis* and *E. granulosus* tapeworm specimens collected in Estonia. Species-specific PCR products for domestic dog (Dog; 56 bp), red fox (Fox; 76 bp), *E. multilocularis* (E. mul; 120 bp), and *E. granulosus* (E. gra; 149 bp) are shown. Lane M, FastRuler Ultra Low Range DNA Ladder (Thermo Scientific, Waltham, MA, USA), DNA fragment sizes are 200, 100 and 50 bp. Lanes Negative 1 and 2, negative controls with no dog or fox DNA added, respectively; lanes Negative 3 and 4, negative controls with no parasite DNA added.

```
                   10        20        30        40        50        60        70
           ....|....|....|....|....|....|....|....|....|....|....|....|....|....|....|.
AB018440   GAATTTATTTTCCGTTAATATTTTGACTCATATATCTAATGTTGCGAAGAGCTGAGTTCTTACCTCAGTAATGTGG
EMest      GAATTTATTTTCCGTTAATATTTTGACTCATATATCTAATGTTGCGAAGAGCTGAGTTCTTACCTCAGTAATGTGG
```

Technical Appendix Figure 2. Nucleotide BLAST (http://blast.ncbi.nlm.nih.gov/Blast.cgi) results for *Echinococcus multilocularis* tapeworm sequence. Alignment of mitochondrial DNA control region sequences (upper panel). AB018440 is a GenBank sequence for *E. multilocularis* tapeworm. EMest is a homologous sequence of *E. multilocularis* tapeworm from 2 red foxes in Tartu, Estonia, and corresponds to positions 8580–8655 in AB018440. Results from sequence similarity search by Nucleotide BLAST (lower panel). Note that the *E. multilocularis* sequence determined from red foxes in Tartu (EMest) shows 100% identity only with a reference sequence for *E. multilocularis* tapeworm (AB018440). For other *Echinococcus* species, the identity is lower (≤93%). Max, maximum; Ident, identity; Accession, GenBank accession number.

Sequences producing significant alignments:

Select: All None Selected:0

Alignments Download GenBank Graphics Distance tree of results

Description	Max score	Total score	Query cover	E value	Ident	Accession
Echinococcus multilocularis mitochondrial DNA, complete genome	141	141	100%	5e-35	100%	AB018440.2
Echinococcus equinus mitochondrion, complete genome	111	111	100%	4e-26	93%	AF346403.1
Echinococcus felidis mitochondrial DNA, complete genome, sample code: EfelUganda	87.9	87.9	100%	6e-19	88%	AB732958.1
Echinococcus granulosus mitochondrial DNA, complete genome, sample code: 52LI07	82.4	82.4	100%	3e-17	87%	AB786664.1
Echinococcus granulosus genotype 1 mitochondrion, complete genome	82.4	82.4	100%	3e-17	87%	AF297617.1

Technical Appendix Figure 3. Sensitivity of PCR for detecting *Echinococcus multilocularis* tapeworms in red fox feces collected in Estonia. PCR amplification of mitochondrial DNA fragments from uninfected red fox fecal samples to which various numbers of *E. multilocularis* eggs were added. Lanes 1, 3, 5, 7, 10, 15 and 20, indicate numbers of *E. multilocularis* eggs added to each sample; lane M, FastRuler Low Range DNA Ladder (Thermo Scientific, Waltham, MA, USA). DNA fragment sizes of the ladder are shown in basepairs above the bands; lane Neg, negative control with no parasite eggs added. Arrows indicate PCR products for *Echinococcus multilocularis* (EM; 120 bp) and red fox (FOX; 76 bp).
